# *MiR-26a* Promotes Ovarian Cancer Proliferation and Tumorigenesis

**DOI:** 10.1371/journal.pone.0086871

**Published:** 2014-01-22

**Authors:** Wenjing Shen, Min Song, Jie Liu, Guangrong Qiu, Tianren Li, Yanjie Hu, Hongbo Liu

**Affiliations:** 1 Department of Gynecology, The First Affiliated Hospital of China Medical University, Shenyang, China; 2 Department of Pathology, The First Affiliated Hospital and College of Basic Medical Sciences, China Medical University, Institute of Pathology and Pathophysiology, Shenyang, China; 3 Experimental Technology Center, China Medical University, Shenyang, China; 4 Department of Medical Genetics, China Medical University, Shenyang, China; 5 Department of Health Statistics, School of Public Health, China Medical University, Shenyang, China; H. Lee Moffitt Cancer Center & Research Institute, United States of America

## Abstract

MicroRNAs (miRNAs) important for posttranscriptional gene expression are involved in the initiation and progression of human cancer. In this study, we reported that *miR-26a* was over-expressed in human EOC specimens and the expression level of extracellular *miR-26a* in plasma can distinguish patients from healthy controls in EOC. Ectopic expression of *miR-26a* in ovarian cancer (OC) cells increased cell proliferation and clonal formation. This growth promoting effect of OC cell growth was mediated by *miR-26a* inhibition of the posttranscription of ER-α. Furthermore, inhibition of *miR-26a* suppressed the tumor formation generated by injecting OC cells in nude mice. Our results suggest that aberrantly expressed *miR-26a* may contribute to OC development.

## Introduction

OC is the fifth most common cancer in women and the leading cause of cancer deaths from gynecological malignancy in western countries [1]. In 2012, there are 22,280 new cases and 15,500 deaths from OC in the United States according to the national cancer statistics. EOC accounts for 85%–90% of ovarian cancer. Unfortunately, the overall prognosis is poor and the molecular events that lead to the development of this disease are still little-known.

MiRNAs represent a large family of endogenous noncoding RNAs and posttranscriptionally regulate gene expression [2]. Recent studies have revealed critical functions of miRNAs in essential processes, including proliferation, differentiation and cell death [3]. Altered expression or mutation of miRNAs has been reported in cancer, such as lung cancer, breast cancer, leukemia and other carcinomas [4]. In lung cancer cells, over-expression of *let-7* inhibited their growth by targeting Ras [5], [6]. Furthermore, *miR-21* directly targets the tumor suppressor PTEN in hepatocellular cancer [7]. Moreover, several studies showed that miRNAs can function either as tumor suppressors (as is the case for the *miR-15a-miR-16-1* cluster [8]) or oncogenes (as is the case for *miR-21* [*9*]).

Genome-wide miRNA expression profiling showed *miR-26a* dysregulation in diverse cancers[10]. In this study, we found that *miR-26a* is over-expressed in human EOC. We demonstrate that inhibition of *miR-26a* decreased proliferation of human EOC cells, and suppressed growth of EOC cells in nude mice. In addition, ERα was down-regulated by *miR-26a* in EOC cells. Furthermore, we found that extracellular *miR-26a* levels in plasma can distinguish patients from healthy controls in EOC. Our study suggests that aberrant expression of *miR-26a* is critical for the development of human EOC and measurement of circulating *miR-26a* may be a good approach to EOC diagnosis.

## Materials and Methods

### Patients

Clinical specimens (including tissue and plasma samples) were collected from patients registered at The First Affiliated Hospital of China Medical University (Shenyang, China). The patients’s information is summarized in Table 1, Table S1 and Table S2.

**Table 1:** clinicopathologic data of ovarian cancer patients.

Total number	26
Pathological tumor stage	
I	3
II	4
III	17
IV	2
Grade	
G1	10
G2	9
G3	7
Histological type	
Serous cystadenocarcinoma	15
Mucinous cystadenocarcinoma	5
Endometrioid carcinoma	3
Clear cell carcinoma	3

### Materials

Antibody against ERα was from Santa Cruz (Santa Cruz, CA, USA), antibody against α-tubulin from Sigma (St Louis, MO, USA). All other reagents were from Sigma. Anti-miR-26a and nonsense of anti-miR were from GenePharma (Shanghai, PR China). Cel-miR-39 mimics were from IBS (Shanghai, PR China).

### *Quantative RT-PCR* (qRT-PCR, Quantitative Reverse Transcriptase-polymerase Chain Reaction)

Total RNA isolated from clinical specimens or cells using Trizol Reagent (Invitrogen, Carlsbad, CA, USA) were reverse-transcribed into cDNA according to the previous report [11]. Isolation of RNA from plasma and quantification of miRNA were carried out as described in [12]. In brief, 25 fmol of Cel-miR-39 mimics was added to 400 ul plasma. Real-time PCR was carried out using SYBR green PCR master mix (TaKaRa, Otus, Shiga, Japan). Amplification and detection were performed using ABI Prism 7700 system (Applied Biosystems, Foster City, CA, USA) according to the manufacturer’s instructions. Cel-miR-39 was taken as reference gene for plasma samples. Primers used were listed in Table S3.

### Cell Culture

The human OC cell lines, SKOV-3, ES2 (Cellbank, Shanghai, PR China) were maintained in McCoy’s 5a supplemented with 10% (v/v) fetal calf serum (Invitrogen, Carlsbad, CA, USA). These cells were incubated at 37°C with 5% CO_2_.

### Vector Construction

Full-length human *miR-26a* was amplified from human genomic DNA and cloned into the pcDNA3.1 at KpnI and XhoI sites according to the previous report [13] and [14]. The human wild-type ERα was generated by PCR from cDNA and the PCR products were inserted into pcDNA3.1 as described in [15].

### Cell Growth Assay

Cell growth was estimated by determination of the cell number and the colony formation. The cells were transfected with *miR-26a* or anti-miR-*26a* using FuGene HD (Roche, Indianapolis, IN) according to the manufacturer’s protocol. After culture for 24 hours the cells were seeded at an initial density of 1×10^5^ per 35 mm-dish. The cells were then harvested at the indicated times and the numbers were counted using the COULTERTM (Beckman, Fullerton, CA, USA).

Cells transfected with *miR-26a* were seeded into 96 well plates at a concentration of 2.5×10^3^ cells, and measured after 48 h using a WST-1 assay (Boster, Wuhan, PR China), performed according to the manufacturer’s protocol.

A total of 500 cells transfected with *miR-26a* or empty vector (Ctrl) were seeded in 35 mm dishes separately in triplicate. Three weeks later, the colonies were fixed with 4% paraformaldehyde, permeated with 20% methanol and stained with crystal violet. The stained cells were eluted by 10% glacial acetic acid and the OD595 values were measured by spectrophotometer as the indicator of cell number.

### Luciferase Reporter Assay

1.5×10^5^ SKOV3 stable cells in 35 mm dishes were co-transfected with pGL3-ERα-WT (1 µg) or pGL3-ERα-Mut (1 µg) and pRL-TK (1 µg) using.

FuGENE® HD Transfection Reagent(Roche, Diagnostics Corp., Indianapolis, IN) following the manufacturer’s protocol. Forty-eight hours after transfection, luciferase activity was measured using a dual luciferase reporter assay system (Promega) and normalized to Renilla luciferase activity.

### Western Blotting

Proteins were separated on SDS–8%PAGE, transferred to PVDF membranes (Amersham, Buckinghamshire, UK) and probed with primary antibodies and secondary antibodies conjugated with horseradish peroxidase. The protein bands were visualized by the Amersham ECL system and scanned. Their densities were determined by ImageQuant 5.2 software (Amersham).

### In vivo Tumorigenesis

SKOV3 cells transfected with *miR-26a* or anti-miR-*26a* separately. Each nude mouse was subcutaneously injected with 2×10^6^ transfected SKOV3 cells. Thirty or thirty-five days after injection the mice were killed and the tumors were taken. The longest and shortest diameter of the tumor was noted as d1 and d2. The tumor volume was calculated using the equation: V = d1×d2×d2/2. Tumor weight was then measured.

### Statistics

Multiple comparisons were assessed by SPSS statistical software for statistical analysis. Wilcoxon Test and Krusikal Wallis H Test were used in statistical analysis of patient samples. Other comparisons between groups for statistical significance were performed with Student’s t test and analysis of variance (ANOVA). Results were considered significant difference at *P<0.05; **P<0.01.

### Ethics Statement

The research meets all requirements for the ethics of experiment. Clinical specimens were collected with consent of patients and healthy volunteers and approval of Medical Research Ethics Committee of the First Affiliated Hospital of China Medical University. All participants have provided their written informed consent to participate in this study. All procedures involving animals were approved by the Institutional Animal Care and Use Committee of China Medical University.

## Results

### Increased Expression of miR-26a in Specimens and Plasma in Human EOC

**Figure 1:**
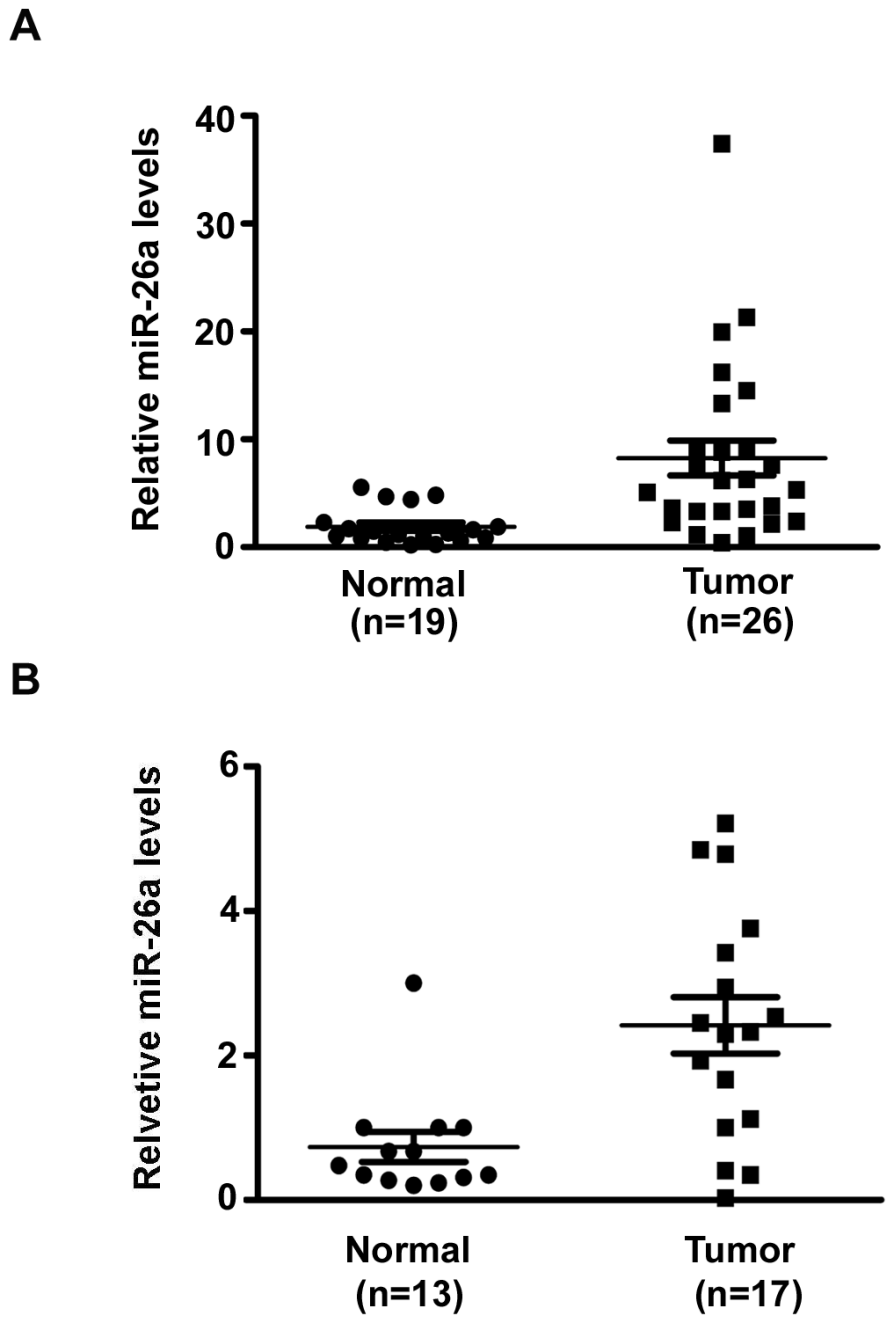
The expression of *miR-26a* was increased in EOC patients. (A)Quantitative analysis of the expression levels of *miR-26a* in EOC samples normalized to those of 18s rRNA by qRT-PCR. Data for each dot were mean value of one sample repeated in three independent experiments (normal, n = 19; tumor, n = 26). **P<0.01 vs Normal. (B)Quantitative analysis of the expression levels of plasma *miR-26a* by qRT-PCR. Data for each dot were mean value of one sample repeated in three independent experiments (normal, n = 13; tumor, n = 17).

It has been observed that the expression level of *miR-26a* was decreased or increased in human malignancies, such as breast carcinoma [16], nasopharyngeal carcinoma [17], [18], and glioblastoma [19], indicating a complicated role of *miR-26a* in progression of the malignancies. To investigate the possible role of *miR-26a* in EOC development, we first examined the expression of *miR-26a* in specimens and plasma in EOC by SYBR-Green stem-loop qRT-PCR [20]. We examined the expression of *miR-26a* in 26 tumor samples and 19 normal ovaries. As shown in Figure 1A, the expression levels of *miR-26a* in tumor samples were much higher than those in normal ovary samples. Similarly, the concentrations of *miR-26a* were higher in plasma from EOC patients (n = 17) than in that from healthy controls (n = 13) (Figure 1B). Together, these results provide us initial evidence that *miR-26a* may play a role in the development of human EOC.

### Over-expressing miR-26a Promoted EOC Cell Growth and Inhibiting miR-26a Suppressed EOC Cell Proliferation

**Figure 2:**
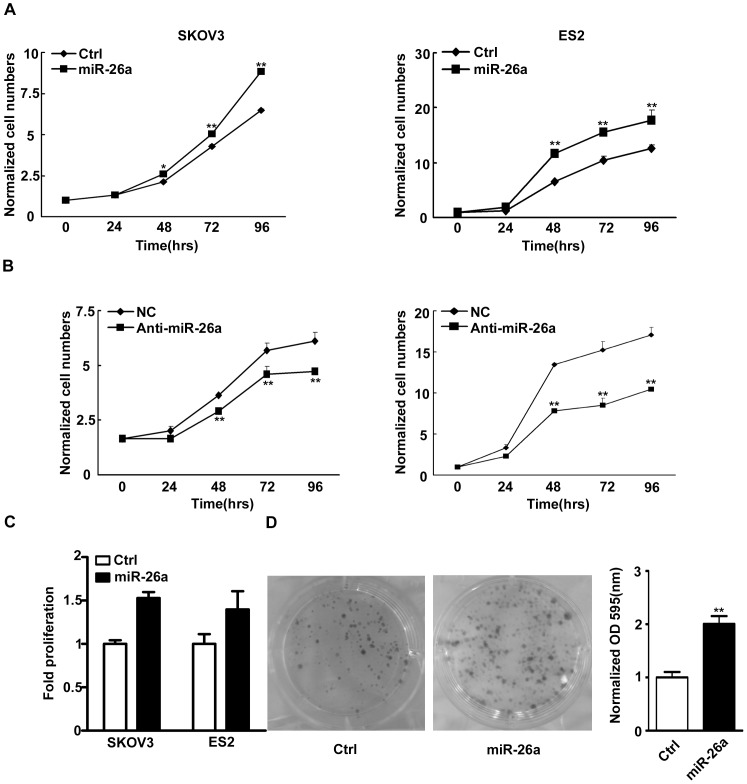
*MiR-26a* promoted EOC cell growth. Growth curves of SKOV3 (A) or ES2 (B) cells transfected with *miR-26a* (left panel) or anti-miR-26a (right panel). Cell numbers were normalized to those in 0 hr. Data were mean±s.e. of three independent experiments in triplicate. *p<0.05, **p<0.01 vs empty vector (Ctrl) or negative control (NC). (C) Cell proliferation transfected with *miR-26a* was measured by a WST-1 assay. Data were mean±s.e. of three independent experiments in triplicate. **p<0.01 vs empty vector (Ctrl) (D) Quantification of the colonies formed by Ctrl or *miR-26a* transfected cells. Data were mean±s.e. of three independent experiments. **P<0.01 vs Ctrl.

We next examined whether *miR-26a* affects EOC cell growth using SKOV3 and ES2 cells as models. As shown in Figure 2A, the growth ability of SKOV3 and ES2 cells was increased by over-expression of *miR-26a*. On the contrary, the proliferation of the cells transfected with anti-miR-26a was markedly decreased compared with that of the cells transfected with nonsense (Figure 2B). WST-1 assay showed similar results in cell proliferation (Figure 2C). These results suggest that *miR-26a* indeed affected EOC cell growth.

The notion was further tested in the clonal formation assay. The number and size of the colonies formed were markedly increased in *miR-26a* transfected cells (Figure 2D). Together, these results suggest that *miR-26a* was indeed involved in the regulation of EOC cell growth.

### ERα was a Target Gene of miR-26a in EOC Cells

**Figure 3:**
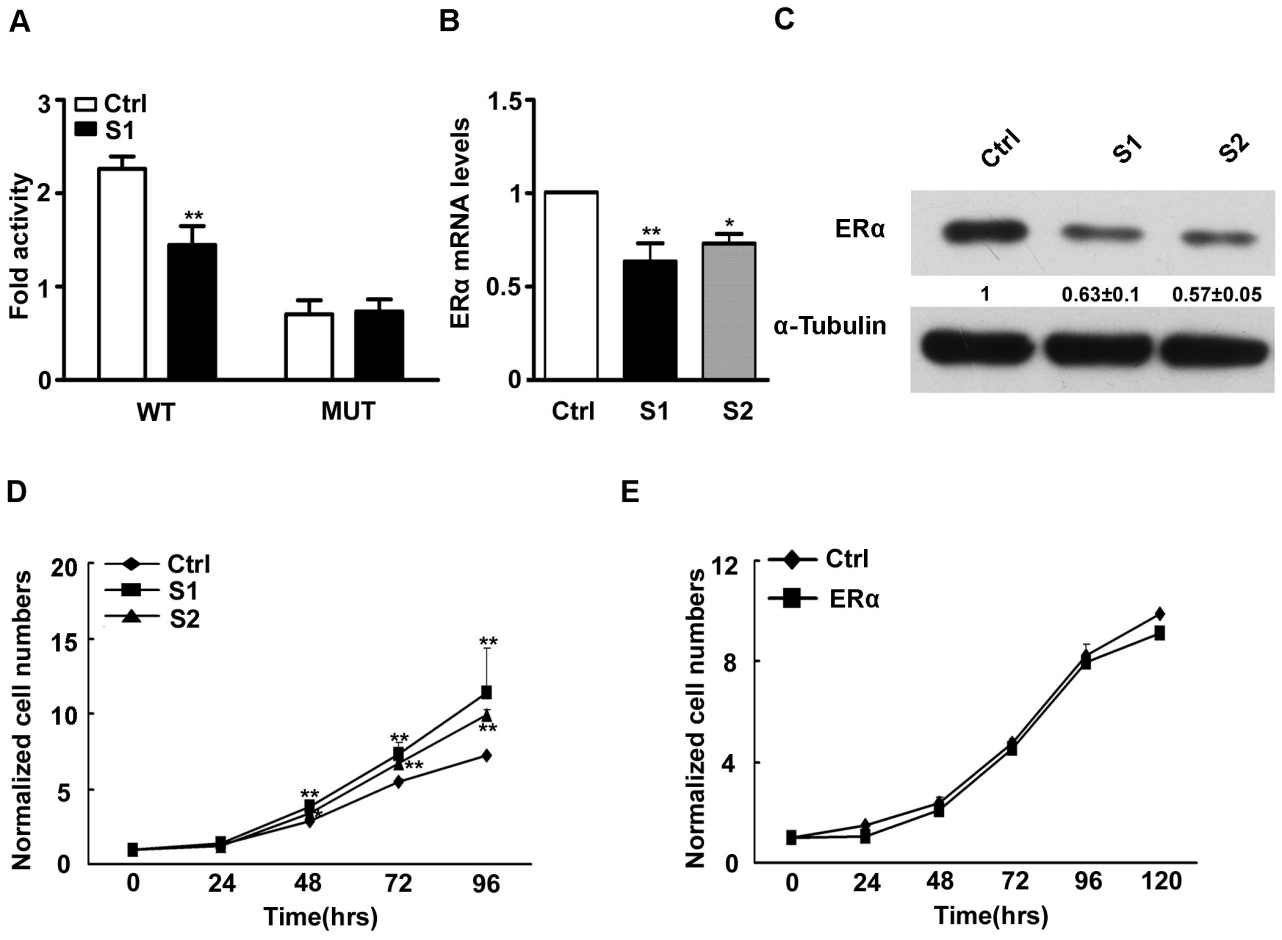
ERα was a target of *miR-26a*. (A) Stable 1 cells (S1) were transfected with pGL3-ERα-WT (WT) reporter vector or pGL3-ERα-Mut (MUT). Data were mean±s.e. of three independent experiments. **P<0.01 vs control cells (Ctrl). (B) Quantitative analysis of the expression levels of ERα in Stable1 (S1), Stable2 (S2) and control (Ctrl) cells were determined and normalized to GAPDH mRNA levels. *p<0.05,**P<0.01 vs Ctrl. (C) Western-blot analysis of total cell lysates extracted from indicated stable cells using the indicated antibodies. Data was representative of three independent experiments and quantitation normalized with the Ctrl. Tubulin served as a loading control. (D) The growth curves of S1, S2 and Ctrl cells. Data were mean±s.e. of three independent experiments in triplicate. *p<0.05, **P<0.01 vs Ctrl. (E) Over-expression of ERα decreased the growth of S1 cells after transfection. Cell numbers were normalized to Ctrl cells. Data were mean±s.e. of three independent experiments in triplicate.

We then investigated the mechanisms by which *miR-26a* promote EOC cell proliferation. In patients with hepatocellular carcinoma, the expression of *miR-26a* was higher in women than in men [21]. Moreover, *miR-26a* regulated liver tumor cell growth by targeting ERα [22]. As shown in Figure 3A, the luciferase activity of pGL3-ERα-WT in SKOV3-stable1 cells (S1) was much lower than in control cells. The luciferase activity of pGL3-ERα-Mut was rescued in Stable1 cells. We next examined whether *miR-26a* could regulate endogenous ERα expression in EOC cells. Compared with control, endogenous ERα mRNA levels (Figure 3B) and protein levels (Figure 3C) were down-regulated when cells were transfected with *miR-26a*.

### MiR-26a Controlled the Proliferation of EOC Cells through ERα

Given the fact that *miR-26a* was involved in proliferation of EOC cells and ERα was a target of *miR-26a*, we next tested whether over-expression of ERα could rescue promotion of proliferation by *miR-26a* in EOC cells. Moreover, stable expression of *miR-26a* in two SKOV3 cell clones (S1 or S2) increased the growth of the cells about 1.58 or 1.37 fold, respectively (Figure 3D). QRT-PCR analysis showed that *miR-26a* expression level was greatly increased in S1 and S2 cells (Figure S1). As shown in Figure 3E, whereas growth of S1 cells transfected with ERα, were not different from that of control cells. These results indicate that *miR-26a* controlled proliferation of EOC cells through regulation of ERα expression.

### MiR-26a Promoted the Development of Tumor in Nude Mice

**Figure 4:**
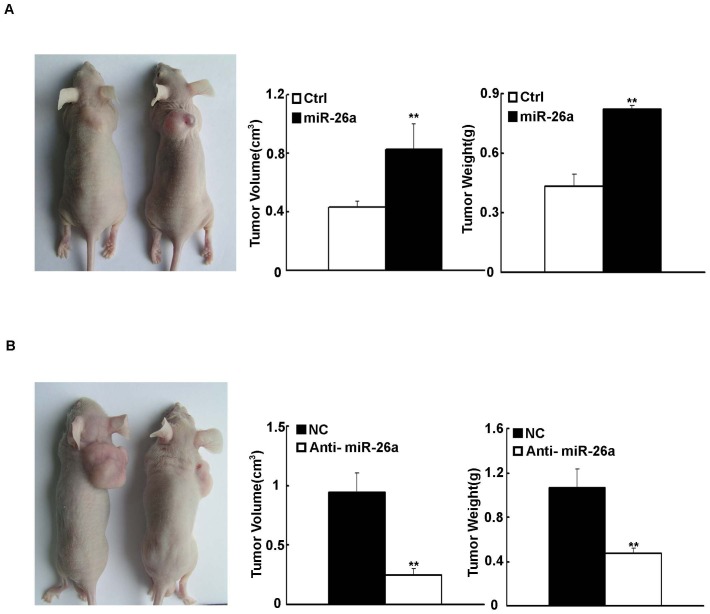
*MiR-26a* promoted the development of tumor in nude mice. Tumor formation generated by the SKOV3 cells. (A) transfected with *miR-26a* or anti-miR-26a(B) Nude mice were subcutaneously injected with 2×10^6^ transfected cells. Representative images, measurement of the final volume and weight of the tumors formed. The tumor sizes were measured and calculated in Materials and methods. Data were mean±s.d. of 4–6 mice. **P<0.01 vs empty vector or nonsense.

To provide direct evidence that *miR-26a* was responsible for EOC development, SKOV3 cells transfected with either *miR-26a* or anti-miR-26a were injected into the flank of nude mice as described. A week after implantation, xenografted tumors could be seen. After thirty or thirty-five days, all nude mice were killed and the tumors were taken out and weighed. At the macroscopic observation, the differences in tumor size and volume among the two groups were indicated. The tumor volume generated from empty vector was 0.435±0.042 (cm^3^, P<0.01), that in *miR-26a* group was 0.829±0.172 (cm^3^, P<0.01) (Figure 4A), that in negative control group was 0.941±0.163 (cm^3^, P<0.01), and that in anti-miR-26a was 0.248±0.05 (cm^3^, P<0.01) (Figure 4B). The average weight of the tumor generated from empty vector was 0.433±0.07, whereas the average weight of the tumor generated from *miR-26a* was 0.821±0.02 (gram, P<0.01). The average weight of the tumor generated from nonsense was 1.062±0.169, whereas the average weight of the tumor generated from anti-miR-26a was 0.473±0.05 (gram, P<0.01), respectively. The results mentioned above suggest that *miR-26a* was critical for the development of EOC *in vivo*.

## Discussion

In this study, we have shown that expression level of *miR-26a* was greatly increased in human EOC samples, and blood-based *miR-26a* level can distinguish patients from healthy controls in EOC. Our results also displayed that expression level of *miR-26a* affected EOC cell growth in culture. Moreover, ERα was a target for *miR-26a* in EOC cells. Furthermore, expression level of *miR-26a* affected tumor formation in nude mice. Therefore, our results are consistent with an idea that *miR-26a* is critical for the development of human EOC.

MicroRNAs are known to regulate the expression of genes involved in several biological processes such as development, proliferation, apoptosis and stress response [23]. Recent studies showed a direct link between miRNAs and human cancers [4], [24], [25], [26]. MiRNAs can be oncogenic miRNA (oncomirs) or tumor suppressor relevant to cancer. As previously shown, loss of oncogenic *miR-21*, acting to repress RHOB expression, is associated with an elevation of RHOB in hepatocellular carcinoma and breast cancer cells [13]. The importance of other miRNAs, such as *let-7*, *miR-16*, *miR-126* and *miR-125b* has also been demonstrated [27]. Recent microarray profile data shows *miR-26a* dysregulation in diverse cancer [4]. In liver cancer, *miR-26a* protects normal liver tissue from hepatocellular carcinoma-promoting inflammation[21], and appears to antagonize human breast cancer [16] and rhabdomyosarcoma [28]. On the contrary, *miR-26a* facilitates glioblastoma formation as an oncogene [19]. Our results obtained from gain-of-function and loss-of-function approaches indicated that *miR-26a* promoted proliferation and inhibition of *miR-26a* suppressed EOC cell proliferation.

In ovarian cancer, ERα expression has been studied to correlate to chlinico-pathological parameters and prognosis [29], [30]. Previous study showed that it did not reveal any correlations with histologic type of tumors and ovarian cancer grading [30]. Univariate survival analysis revealed that patients with positive-ERα status had a significant better progression-free survival compared with the patients with no expression[31]. In our results, the expression of *miR-26a* in specimens and plasma in EOC were much higher than those in normal ovary samples, and *miR-26a* promote EOC cell proliferation by targeting ERα. It means that *miR-26a* might be an important factor in the survival of ovarian cancer.

Circulating biomarkers are used to diagnostic disease, monitor therapeutic effect and predict recurrence in clinical applications. The discovery of circulating miRNAs in cancer patients indicates their potential application as powerful biomarkers in cancer diagnostics[32]. Recent studies revealed that cancer-related miRNAs could stably detectable in plasma and serum which originate from cancer tissues [4], [33]. Our results investigate the possibility of *miR-26a* applied as a biomarker in clinical setting through examining the expression of *miR-26a* in plasma of EOC patients.

In conclusion, inhibition of *miR-26a* decreased EOC cell growth in culture and in nude mice. *MiR-26a* affected proliferation of EOC cells by targeting ERα. An important implication of current study is that *miR-26a* might be a potential target for therapeutic intervention to human EOC.

## Supporting Information

Figure S1 ***MiR-26a*** **expression level was greatly increased in S1 and S2 cells.** QRT-PCR determined the expression levels of *miR-26a* in S1 and S2 cells. Data were mean±s.e. of three independent experiments in triplicate. **P<0.01 vs Ctrl.
(DOC)

Table S1 **clinicopathologic data and** ***miR-26a*** **expression level of control.**
(DOC)

Table S2 **clinicopathologic data and** ***miR-26a*** **expression level of ovarian cancer patients.**
(DOC)

Table S3 **Primers used in Quantative RT-PCR.**
(DOC)
