# Supplementary material for: MiR-26a Promotes Ovarian Cancer Proliferation and Tumorigenesis
Source: PLoS One. 2014 Jan 22;9(1):e86871. doi: 10.1371/journal.pone.0086871 (PMC3899311; doi:10.1371/journal.pone.0086871)
Supplement: Table S1 — clinicopathologic data and miR-26a expression level of control. (DOC) [file pone.0086871.s002.doc]

**Table S1:** [**clinicopathologic data**](app:ds:clinicopathologic data) **and *miR-26a* expression level of control**

| control  number(19) | *miR-26a* expression level | age | diagnosis |
| --- | --- | --- | --- |
| 1 | 1.19 | 64 | myoma |
| 2 | 4.41 | 48 | myoma |
| 3 | 4.82 | 48 | myoma |
| 4 | 5.56 | 51 | myoma |
| 5 | 1.70 | 51 | myoma |
| 6 | 0.24 | 46 | myoma |
| 7 | 1.32 | 46 | myoma |
| 8 | 4.68 | 46 | myoma |
| 9 | 1.49 | 49 | myoma |
| 10 | 0.58 | 54 | myoma |
| 11 | 0.46 | 54 | myoma |
| 12 | 0.85 | 54 | myoma |
| 13 | 1.00 | 51 | myoma |
| 14 | 1.00 | 51 | myoma |
| 15 | 1.60 | 53 | myoma |
| 16 | 1.87 | 53 | myoma |
| 17 | 2.30 | 53 | myoma |
| 18 | 0.82 | 52 | myoma |
| 19 | 0.21 | 48 | myoma |
