# Supplementary material for: MiR-26a Promotes Ovarian Cancer Proliferation and Tumorigenesis
Source: PLoS One. 2014 Jan 22;9(1):e86871. doi: 10.1371/journal.pone.0086871 (PMC3899311; doi:10.1371/journal.pone.0086871)
Supplement: Table S2 — clinicopathologic data and miR-26a expression level of ovarian cancer patients. (DOC) [file pone.0086871.s003.doc]

**Table S2:** [**clinicopathologic data**](app:ds:clinicopathologic data) **and *miR-26a* expression level of ovarian cancer patients**

| ovarian cancer case(26) | *miR-26a* expression level | histology type | grade | stage | age |
| --- | --- | --- | --- | --- | --- |
| 1 | 5.09 | clear cell carcinoma | G3 | Ic | 60 |
| 2 | 21.33 | serous cystadenocarcinoma | G2 | Ⅲc | 53 |
| 3 | 3.53 | serous cystadenocarcinoma | G3 | Ⅲc | 73 |
| 4 | 13.33 | serous cystadenocarcinoma | G2 | Ⅲc | 51 |
| 5 | 1.05 | mucinous cystadenocarcinoma | G1 | IV | 52 |
| 6 | 1.15 | mucinous cystadenocarcinoma | G2 | Ⅲc | 55 |
| 7 | 2.29 | serous cystadenocarcinoma | G2 | Ⅲc | 59 |
| 8 | 3.32 | endometrioid carcinoma | G1 | Ⅱb | 44 |
| 9 | 6.27 | clear cell carcinoma | G1 | Ⅲb | 64 |
| 10 | 2.14 | mucinous cystadenocarcinoma | G3 | Ⅰa | 54 |
| 11 | 8.81 | mucinous cystadenocarcinoma | G1 | Ⅲc | 59 |
| 12 | 3.81 | serous cystadenocarcinoma | G2 | Ⅲc | 56 |
| 13 | 2.37 | serous cystadenocarcinoma | G2 | Ⅲc | 57 |
| 14 | 19.94 | endometrioid carcinoma | G1 | Ⅲc | 45 |
| 15 | 16.23 | endometrioid carcinoma | G1 | Ⅲc | 52 |
| 16 | 14.51 | mucinous cystadenocarcinoma | G3 | IIIa | 48 |
| 17 | 0.40 | clear cell carcinoma | G1 | IIc | 59 |
| 18 | 6.19 | serous cystadenocarcinoma | G3 | Ⅲc | 56 |
| 19 | 5.33 | serous cystadenocarcinoma | G1 | IV | 61 |
| 20 | 9.18 | serous cystadenocarcinoma | G3 | Ⅲc | 59 |
| 21 | 37.43 | serous cystadenocarcinoma | G2 | Ⅰa | 65 |
| 22 | 7.25 | serous cystadenocarcinoma | G2 | Ⅲc | 49 |
| 23 | 3.32 | serous cystadenocarcinoma | G1 | IIc | 53 |
| 24 | 9.15 | serous cystadenocarcinoma | G3 | Ⅲc | 55 |
| 25 | 7.62 | serous cystadenocarcinoma | G1 | IIb | 52 |
| 26 | 3.61 | serous cystadenocarcinoma | G2 | Ⅲc | 59 |
