# Supplementary material for: MiR-26a Promotes Ovarian Cancer Proliferation and Tumorigenesis
Source: PLoS One. 2014 Jan 22;9(1):e86871. doi: 10.1371/journal.pone.0086871 (PMC3899311; doi:10.1371/journal.pone.0086871)
Supplement: Table S3 — Primers used in Quantative RT-PCR. (DOC) [file pone.0086871.s004.doc]

**Table S3: Primers used in Quantative RT-PCR**

| **List of primers used in this study. (5`-3`)** | |
| --- | --- |
| *MiR-26a* RT primer | gttggctctggtgcagggtccgaggtattcgcaccagagccaacagccta |
| *MiR-26a* ST forward primer | ccggcgttcaagtaatccagg |
| ST reverse primer | gtgcagggtccgaggt |
| 18s rRNA-sense | cgccgctagaggtgaaattc |
| 18s rRNA-antisense | ttggcaaatgctttcgctc |
| GAPDH-sense | tgcaccaccaactgcttag |
| GAPDH-antisense | gacgcagggatgatgttc |
| ERα-sense | gtgggatacgaaaagaccgaaga |
| ERα-antisense | ggggcagctctcatgtctc |
